# Cannabis Use and CKD: Epidemiological Associations and Mendelian Randomization

**DOI:** 10.1016/j.xkme.2022.100582

**Published:** 2022-12-11

**Authors:** Sergio Dellepiane, Ishan Paranjpe, Madhumitha Rajagopal, Samir Kamat, Ross O’Hagan, Faris Gulamali, Joshua L. Rein, Alexander W. Charney, Ron Do, Steven Coca, Benjamin S. Glicksberg, Girish N. Nadkarni

**Affiliations:** 1 Mount Sinai Clinical Intelligence Center (MSCIC), Icahn School of Medicine at Mount Sinai, New York, NY; 2 Hasso Plattner Institute for Digital Health at Mount Sinai, Icahn School of Medicine at Mount Sinai, New York, NY; 3 Department of Medicine, Division of Nephrology, Icahn School of Medicine at Mount Sinai, New York, NY; 4 Department of Genetics and Genomic Sciences, Icahn School of Medicine at Mount Sinai, New York, NY; 5 Charles Bronfman Institute for Personalized Medicine, Icahn School of Medicine at Mount Sinai, New York, NY; 6 Pamela Sklar Division of Psychiatric Genomics, Icahn School of Medicine at Mount Sinai, New York, NY; 7 Department of Psychiatry, Icahn School of Medicine at Mount Sinai, New York, NY; 8 Department of Medicine, Division of Data Driven and Precision Medicine, Icahn School of Medicine at Mount Sinai, New York, NY

**Keywords:** Cannabis, chronic kidney disease, Mendelian randomization

## Abstract

**Rationale & Objective:**

The association between cannabis use and chronic kidney disease (CKD) is controversial. We aimed to assess association of CKD with cannabis use in a large cohort study and then assess causality using Mendelian randomization with a genome-wide association study (GWAS).

**Study Design:**

Retrospective cohort study and genome-wide association study.

**Setting & Participants:**

The retrospective study was conducted on the All of Us cohort (N=223,354). Genetic instruments for cannabis use disorder were identified from 3 GWAS: the Psychiatric Genomics Consortium Substance Use Disorders, iPSYCH, and deCODE (N=384,032). Association between genetic instruments and CKD was investigated in the CKDGen GWAS (N > 1.2 million).

**Exposure:**

Cannabis consumption.

**Outcomes:**

CKD outcomes included: cystatin-C and creatinine-based kidney function, proteinuria, and blood urea nitrogen.

**Analytical Approach:**

We conducted association analyses to test for frequency of cannabis use and CKD. To evaluate causality, we performed a 2-sample Mendelian randomization.

**Results:**

In the retrospective study, compared to former users, less than monthly (OR, 1.01; 95% CI, 0.87-1.18; *P* = 0.87) and monthly cannabis users (OR, 1.15; 95% CI, 0.86-1.52; *P* = 0.33) did not have higher CKD odds. Conversely, weekly (OR, 1.28; 95% CI, 1.01-1.60; *P* = 0.04) and daily use (OR, 1.25; 95% CI, 1.04-1.50; *P* = 0.02) was significantly associated with CKD, adjusted for multiple confounders. In Mendelian randomization, genetic liability to cannabis use disorder was not associated with increased odds for CKD (OR, 1.00; 95% CI, 0.99-1.01; *P* = 0.96). These results were robust across different Mendelian randomization techniques and multiple kidney traits.

**Limitations:**

Likely underreporting of cannabis use. In Mendelian randomization, genetic instruments were identified in the GWAS that included individuals primarily of European ancestry.

**Conclusions:**

Despite the epidemiological association between cannabis use and CKD, there was no evidence of a causal effect, indicating confounding in observational studies.

## Graphical abstract

**Figure:**
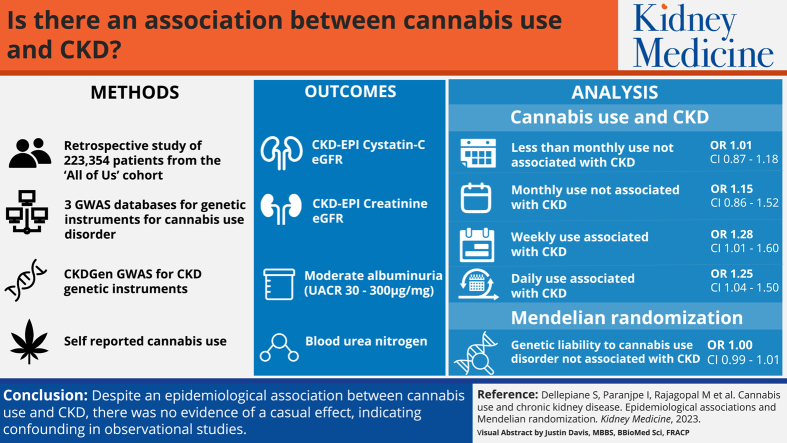

**Plain-Language Summary**

The association between cannabis use and chronic kidney disease (CKD) is controversial. Previous studies have been limited by several biases including underreporting. We used Mendelian randomization, a method designed to simulate a randomized study, to investigate the association between cannabis use and CKD. We found limited evidence that cannabis use causes CKD, which has implications for health policy.

According to the National Survey on Drug Use and Health, half of adults in the United States have used cannabinoid-containing products at least once, either for recreational or medical purposes.^1^ In parallel, cannabis use is being increasingly legalized worldwide, and different cannabinoids have been approved by different pharmacological agencies to treat pain, nausea, anorexia, and other medical conditions.^2^ As such, cannabinoid consumption is expected to increase.^3^ However, it is still unclear whether there is a causal relationship between cannabinoid consumption and different health outcomes, including chronic kidney disease (CKD).

The cannabinoid receptors (G protein-coupled receptors CB1 and CB2) are ubiquitous in mammals. In the kidney, CB1 and CB2 regulate renal blood flow, glomerular filtration rate (GFR), glomerular permeability, interstitial collagen production, and tubular function.^4^ In preclinical investigations, CB1 has been associated with kidney disease progression, whereas CB2 is involved in tubular cell turnover (both reparative and detrimental).^3^^,^^5^, ^6^, ^7^ However, the assessment of cannabis effects on kidney function is challenging because of the quality of available data (most often self-reported surveys) and the proven association among cannabis consumption and multiple comorbid conditions^8^ and socioeconomic confounding factors.^9^^,^^10^

Mendelian randomization allows the estimation of a causal effect from observational data in the presence of confounding factors.^11^ The first step of Mendelian randomization consists in using genome-wide association studies (GWAS) to identify allelic variants associated with the exposure (ie, cannabis consumption), with dependence on certain assumptions.^12^ The second step consists in estimating the risk of outcome in individuals with and without the identified variants. This analysis can be performed using data from the same (1-sample Mendelian randomization) or a different GWAS (2-sample Mendelian randomization).^13^ Because population alleles are distributed randomly and independently from environmental confounders, Mendelian randomization mimics a randomized clinical trial in which individuals at high versus low risk of exposure are compared in relation to the investigated outcome.

To address the relationship between cannabis use, its frequency, and kidney disease, we performed the largest observational data analysis to date using a large, public, and nationally representative cohort; subsequently, we used a 2-sample Mendelian randomization to assess causality.

## Methods

### Observational Study

We investigated the association between cannabis use and CKD by using data from All of Us cohort,^14^ an NIH-funded project aimed to collect and prospectively follow health data from 1 million adults across the United States (https://allofus.nih.gov/). This large observational cohort includes data from 340 sites across the United States and is ethnically, socially, and geographically diverse. Electronic health records (EHRs), health questionnaires, physical measurements, and wrist accelerometer data are included. There are no exclusion criteria, and participation is voluntary. Informed consent was obtained as part of the All of Us recruitment process. The All of Us study protocol was approved by the All of Us Institutional Review Board (protocol no. 11-01139).

We used EHR data that was transformed to Observational Medical Outcomes Partnership standard vocabulary (Systematized Nomenclature of Medicine Clinical Terms [SNOMED]). History of hypertension (SNOMED: 320128), type 2 diabetes (SNOMED: 201826), hyperlipidemia (SNOMED: 432867), and coronary artery disease (SNOMED: 53741008) were determined by the presence of SNOMED terms in the EHR. For consistency with the Mendelian randomization analysis (see below), CKD was also defined with SNOMED (codes 46271022 and 193782). Age and sex at birth were taken from questionnaires at the time of enrollment. Median body mass index values from all available measurements in the EHR were computed for each participant.

Surveys that included information on demographics, lifestyle, substance use, and personal and family history were administered at the time of enrollment in the cohort (the questions about cannabis use are listed in Item S1).

We included in our analyses the participants who completed the lifestyle survey; in particular, we excluded those who did not answer the questions about substance use, insurance, and education. Because lifetime cannabis use may identify minimal and remote exposure, we performed a further analysis based on cannabis use frequency. Participants who responded to the use frequency question were divided based on the previous 3-month consumption: no users, and current users who used cannabis less than monthly, monthly, weekly, and daily (summary statistics are reported in Table S1).

The association between cannabis use frequency and CKD in All of Us was estimated by fitting a logistic regression model adjusted for age, sex at birth, body mass index (in kg/m^2^), race, education level, presence of health insurance, cigarette smoking (>100 cigarettes smoked in lifetime^15^), and history of hypertension, hyperlipidemia, and type 2 diabetes (same SNOMED codes as above). Cardiovascular risk factors were included in the model to account for potential confounding because these are risk factors for CKD and may also be affected by cannabis use. Health insurance and education level were chosen as measures of socioeconomic status and adjusted for given the association of CKD and lower socioeconomic status.^16^ All analyses were performed using R version 4.0.3.^17^ A *P* value < 0.05 was considered significant.

### Mendelian Randomization

We used a 2-sample Mendelian randomization study to assess causal association between cannabis use and CKD. In the first step, genetic variants associated with cannabis consumption were identified in a GWAS for substance abuse disorder (exposure GWAS). These variants are called *genetic instruments*. In the second step, individuals with and without the identified variants were compared to a GWAS of CKD (outcome GWAS). Mendelian randomization mimics a randomized controlled trial, as the presence of high risk variants serves as a proxy for an intervention, and individuals with high versus low risk of exposure (cannabis use) are compared in rapport to an outcome (CKD).

### Cannabis Use Genetic Instrument Selection

We included single-nucleotide polymorphisms (SNPs) significantly associated with cannabis use disorder (CUD). Instruments for CUD were obtained from the largest available GWAS meta-analysis, which includes 3 cohorts^18^: the Psychiatric Genomics Consortium Substance Use Disorders working group, iPSYCH, and deCODE. This pooled GWAS included 20,916 cases and 363,116 controls. Cases met the Diagnostic and Statistical Manual of Mental Disorders (Third Edition Revised), Diagnostic and Statistical Manual of Mental Disorders (Fourth Edition), or Diagnostic and Statistical Manual of Mental Disorders (Fifth Edition) criteria for CUD or had an *International Classification of Diseases, Tenth Revision* code for cannabis abuse or dependence. For the main analysis, we used 32 SNPs as instrumental variables that were associated with CUD (*P* < 5 × 10^-6^), clumped at linkage disequilibrium r^2^ = 0.001, and within 10 Mb. We included 2 genome-wide significant SNPs (*P* < 5 × 10^-8^) in a secondary analysis. SNP effect sizes for the Mendelian randomization analysis were obtained from the CUD-pooled GWAS.

### CKD Outcome

Summary statistics from CKD GWAS performed by the CKDGen consortium were obtained.^19^^,^^20^ The CKDGen consortium includes different GWAS of kidney traits and has data from >1.2 million individuals. Different outcome GWAS were used depending on the outcome variable; sample size varied between 348,954 and 765,000.^19^, ^20^, ^21^. The dichotomous outcomes were: CKD defined as creatinine estimated GFR (eGFR) <60 mL/min (Chronic Kidney Disease Epidemiology Collaboration [CKD-EPI] creatinine formula), and microalbuminuria defined as urinary albumin-creatinine ratio between 30 and 300 μg/mg. Continuous outcomes included CKD-EPI creatinine-based GFR (2012 formula), CKD-EPI cystatin-C–based GFR (2012 formula), blood urea nitrogen (BUN) levels, and urinary albumin-creatinine ratio.^22^ SNP effect sizes for the CKD outcomes were obtained from the CKD GWAS from the CKDGen consortium.

### Mendelian Randomization for CKD and Kidney Traits

For the primary analysis, the effects of genetic instruments for CUD on CKD and kidney traits were estimated using the Mendelian randomization robust adjusted profile score^23^ that is robust to weak instruments. All analyses were performed using methods implemented in the TwoSampleMR package.^24^^,^^25^ Sensitivity analyses to identify violations of the instrument variable assumptions were performed using the fixed-effects inverse weighted, maximum likelihood, weighted median, and penalized weighted median methods. Leave-one-out analysis was also performed to assess for outliers that may be driving the observed exposure-outcome relationship. The MR-PRESSO global test^11^ was performed to test for evidence of horizontal pleiotropy. A power calculation was performed using mRnd.^26^ For all Mendelian randomization analyses, statistical significance was set at *P* < 0.05.

## Results

### Observational Association of Cannabis Use and CKD

**Figure 1:**
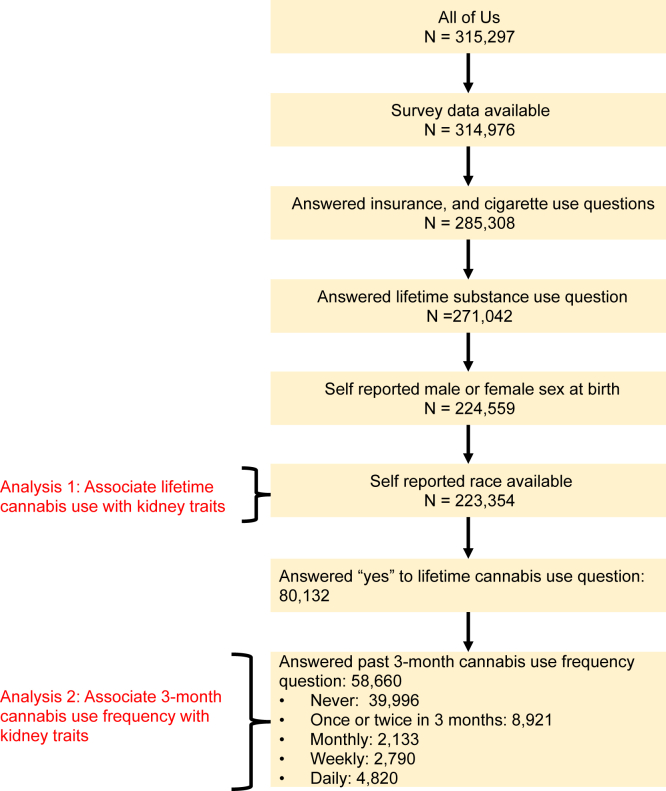
**“**All of Us” cohort overview. Flowchart describes the preprocessing steps used to generate the final subset of the All of Us cohort used.

**Figure 2:**
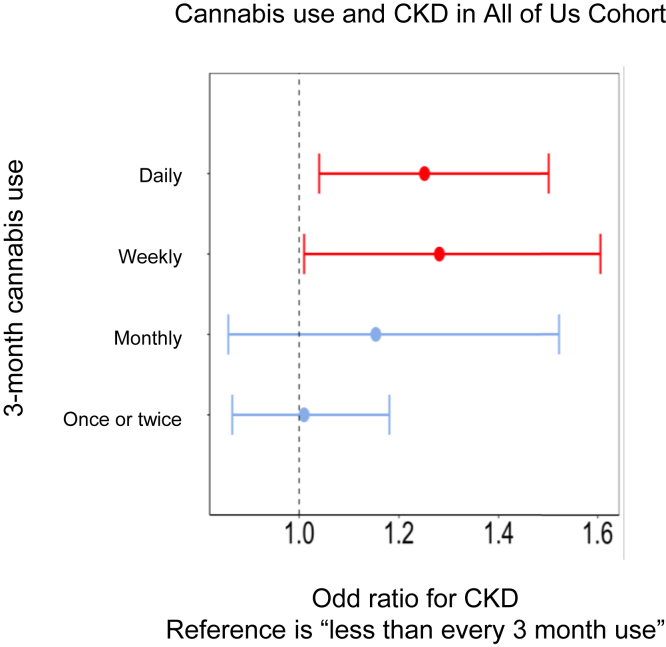
Odds ratio for chronic kidney disease (CKD) across different levels of cannabis consumption.

At the time of our analysis, the All of Us cohort included data from 315,297 participants. EHR data was available for 227,740 individuals. We excluded individuals with missing self-reported race or sex at birth or an incomplete cannabis consumption survey, and we identified 223,354 individuals eligible for the analysis (Fig 1). Demographic and main clinical variables are reported in Table 1. Lifetime cannabis use (n=80,132) was not significantly associated with CKD adjusted for adjusted for age, sex at birth, body mass index, race, insurance status, cigarette smoking, and history of hypertension, hyperlipidemia, and type 2 diabetes (odds ratio [OR], 1.05; 95% confidence interval [CI], 0.94-1.17; *P* = 0.38). Compared to former users, less than monthly (OR, 1.01; 95% CI, 0.87-1.18; *P* = 0.87) and monthly cannabis users (OR, 1.15; 95% CI, 0.86-1.52; *P* = 0.33) did not have higher odds of prevalent CKD. Conversely, weekly (OR, 1.28; 95% CI, 1.01-1.60; *P* = 0.04) and daily use (OR, 1.25; 95% CI, 1.04-1.50; *P* = 0.02) were significantly associated with prevalent CKD (Fig 2). All analyses were adjusted for age, sex at birth, body mass index, race, insurance status, education level, cigarette smoking, and history of hypertension, hyperlipidemia, and type 2 diabetes.

**Table 1:** All of US Cohort Summary (Lifetime Cannabis Use)

Variable	Lifetime Cannabis Use		
	No (N=144,477)	Yes (N=80,132)	*P*
Sex at birth, N (%)			<0.001
Male	57,400 (40%)	31,299 (39%)	
Race, N (%)			<0.001
Asian	6,894 (4.8%)	2,243 (2.8%)	
Black or African American	35,574 (25%)	20,171 (25%)	
More than 1 population	2,782 (1.9%)	2,087 (2.6%)	
Other	3,182 (2.2%)	1,476 (1.8%)	
White	96,045 (66%)	54,155 (68%)	
Age (y), Median (IQR)	59 (43, 69)	54 (37, 67)	<0.001
BMI (KG/M^2^), median (IQR)	28 (24, 34)	28 (24, 33)	<0.001
Chronic kidney disease, n (%)	6,225 (4.3%)	2,633 (3.3%)	<0.001
Hypertension, n (%)	43,713 (30%)	19,934 (25%)	<0.001
Type 2 diabetes, n (%)	7,630 (5.4%)	3,225 (4.1%)	<0.001
Coronary artery disease, n (%)	7,789 (5.4%)	3,282 (4.1%)	<0.001
Hyperlipidemia, n (%)	11,411 (7.9%)	4,803 (6.0%)	<0.001
> 100 Cigarettes smoked in lifetime, n (%)			<0.001
Yes	58,766 (41%)	37,795 (47%)	
Has health insurance			0.05
Yes	136,172 (94%)	75,361 (94%)	

*Note*: Demographics and clinical history summarized by lifetime cannabis use in the All of US cohort are provided. Individuals who answered, “Prefer not to answer” or who skipped the substance use question in the survey were excluded. Sex at birth, age, BMI, and cigarette use were ascertained using physical measurements and surveys administered at the time of enrollment in All of Us. Medical history was obtained from EHR data as well as self-reported survey data.

Abbreviations: BMI, body mass index; EHR, electronic health record; IQR, interquartile range.

### Selection of Genetic Instruments

Table S2 reports the SNPs selected as genetic instruments. Only 2 independent SNPs were significantly associated with CUD in the largest GWAS meta-analysis available.^18^ We used a liberal SNP selection strategy as previously described.^27^ To accommodate SNPs with weak individual association, we applied Mendelian randomization robust adjusted profile scores^23^; 32 SNPs with *P* < 5 × 10^-6^ were used and clumped if r^2^ > 0.001 or they were located within 10 Mb of each other. We also performed the analysis using only the 2 SNPs that met genome-wide significance for CUD. In both analyses, all SNPs had an F statistic that was greater than 10.

### Mendelian Randomization of CUD and CKD

We performed 2-sample Mendelian randomization to assess the causal association of CUD with CKD and other clinically relevant kidney traits. In a Mendelian randomization power calculation,^26^ we estimated the power to detect a significant association between significant cannabis use (daily use) and CKD to be 0.99. In this analysis, we used a variance explained of 0.1 as previously reported in the CUD GWAS^18^ and an effect size of 1.25 (OR) as this was the strength of association between daily cannabis use and CKD in our observational analysis. The 32 SNPs used as genetic instrument variables suggested a lack of a causal association with CKD in Mendelian randomization robust adjusted profile score analysis (OR, 1.00; 95% CI, 0.99-1.01; *P* = 0.96). We did not detect horizontal pleiotropy for the CUD–CKD pathway using the MR-PRESSO global test (*P* = 0.26). The lack of association of CUD with CKD remained robust using multiple Mendelian randomization methods, including fixed-effects inverse variance weighted, maximum likelihood, weighted median, and penalized weighted median (Table 2). No evidence of outliers or bias was detected in leave-one-out analysis as all associations remained significant on sequentially excluding each SNP (Fig S1). The association was also not significant using the CUD genome-wide significant SNPs as instrument variables (Table 2).

**Table 2:** MR Estimates for All Kidney Outcomes

MR Association			
Outcome	Method	β (SE) or OR (95% CI)	*P*
eGFR (creatinine)	Inverse variance weighted (fixed effects)	8.7E-4 (6.0E-4)	0.14
eGFR (creatinine)	Maximum likelihood	9.3E-4 (6.4E-4)	0.15
eGFR (creatinine)	Weighted median	3.0E-4 (9.1E-4)	0.75
eGFR (creatinine)	Penalized weighted median	2.8E-4 (9.3E-4)	0.76
eGFR (creatinine)	MR-RAPS	1.0E-3 (6.0E-4)	0.09
eGFR (cystatin-C)	Inverse variance weighted (fixed effects)	0.003 (0.001)	0.002
eGFR (cystatin-C)	Maximum likelihood	0.003 (0.001)	0.001
eGFR (cystatin-C)	Weighted median	0.003 (0.002)	0.27
eGFR (cystatin-C)	Penalized weighted median	0.003 (0.002)	0.03
eGFR (cystatin-C)	MR-RAPS	0.003 (0.001)	0.001
BUN	Inverse variance weighted (fixed effects)	0.005 (0.001)	<0.001
BUN	Maximum likelihood	0.005 (0.001)	<0.001
BUN	Weighted median	0.002 (0.002)	0.15
BUN	Penalized weighted median	0.002 (0.002)	0.22
BUN	MR-RAPS	0.005 (0.001)	<0.001
CKD	Inverse variance weighted (fixed effects)	1.00 (0.99-1.01)	0.96
CKD	Maximum likelihood	1.00 (0.99-1.01)	0.96
CKD	Weighted median	1.00 (0.99-1.01)	0.79
CKD	Penalized weighted median	1.00 (0.99-1.01)	0.79
CKD	MR-RAPS	1.00 (0.99-1.01)	0.96
UACR	Inverse variance weighted (fixed effects)	-0.005 (0.004)	0.25
UACR	Maximum likelihood	-0.005 (0.005)	0.25
UACR	Weighted median	0.002 (0.007)	0.73
UACR	Penalized weighted median	0.003 (0.007)	0.7
UACR	MR-RAPS	-0.005 (0.004)	0.22
MA	Inverse variance weighted (fixed effects)	0.99 (0.96-1.01)	0.27
MA	Maximum likelihood	0.99 (0.95-1.01)	0.27
MA	Weighted median	1.00 (0.96-1.04)	0.92
MA	Penalized weighted median	1.00 (0.96-1.04)	0.86
MA	MR-RAPS	0.98 (0.95-1.01)	0.26

*Note*: The exposure was risk of frequent cannabis use. The outcomes were: estimated glomerular filtration rate (eGFR in mL/min) calculated with both creatinine (unit: mg/dL) and cystatin-C (unit: mg/dL), blood urea nitrogen (BUN in mg/dL), CKD considered as dichotomous outcome (eGFR creatinine < 60 mL/min), urinary albumin-creatinine ratio (UACR in μg/mg), and microalbuminuria (MA) considered as dichotomous outcome (30 < UACR < 300 μg/mg). eGFR outcomes were log transformed before Mendelian randomization analysis and thus β coefficients are reported on a log scale. β coefficients can be interpreted as a unit change in each outcome variable with respect to CUD controls. The odds ratio and 95% confidence interval are provided for dichotomous outcomes (CKD and MA). All genome-wide association studies for eGFR and BUN used log-transformed values as the outcome measure and thus, β coefficients are reported on a log scale.

Abbreviations: BUN, blood urea nitrogen; CI, confidence interval; CKD, chronic kidney disease; CUD, cannabis use disorder; eGFR, estimated glomerular filtration rate; MA, microalbuminuria; MR, Mendelian randomization; MR-RAPS, Mendelian randomization robust adjusted profile score; OR, odds ratio; SE, standard error; UACR, urinary albumin-creatinine ratio.

We then tested for several kidney traits. No causal relationship was found between CUD and urinary albumin-creatinine ratio, microalbuminuria, and eGFR (cystatin-C and creatinine). However, when we validated our findings by using multiple Mendelian randomization methods, BUN and eGFR (cystatin-C) returned a significant association with CUD (*P* < 0.05), although the effect size was clinically insignificant.

## Discussion

Assessing the risk of kidney disease in cannabis users is highly relevant. Cannabis is among the most consumed recreational drugs worldwide; according to the National Survey on Drug Use and Health, half of the US population has used it at least once in their lifetime.^1^ On the other hand, cannabinoids have been approved by several pharmacological agencies (including the Food and Drug Administration and European Medical Agency) for the treatment of symptoms commonly experienced by people living with CKD (eg, nausea, anorexia, pruritus).^3^

Consumption of cannabis derivatives has been inconsistently associated with CKD in observational studies.^4^^,^^28^^,^^29^ To assess the possible relationship between cannabis and CKD, we at first performed the largest observational study to date: among >220,000 patients with valid data, lifetime cannabis use was not associated with higher risk of kidney disease; however, daily and weekly cannabis consumption was significantly associated with CKD. Thus, to assess causality, we performed a Mendelian randomization analysis. In our analysis, we did not find any causal relationship between cannabis use and CKD. This was robust with different CKD-related outcomes (eg, GFR and proteinuria).

Previous literature has been conflicted about this relationship and is affected by multiple biases. Lu et al^28^ performed a cross-sectional study on this topic using data from 13,995 US adults. In the unadjusted analysis, cannabis was significantly associated with lower eGFR. After adjusting for different variables (age, sex, self-reported race, education level, marital status, income level, alcohol consumption, cigarette smoking, hypertension status, diabetes status, and mean systolic blood pressure), the association was lost, but serum creatinine had an increasing trend when nonpast- and current-users were compared. The trend was not significant but persisted also when the patients were stratified based on the presence of cardiovascular disease. In a study on 1,600 adults, persistent cannabis users (n=114) had faster GFR decline over a follow-up of 4.1 years.^30^ The analysis was limited by the small sample size and the presence of multiple confounding factors (higher prevalence of male sex and heavy tobacco consumption in cannabis users) but was based on prospective data and had better quantification of cannabis use compared to Lu et al investigation. Ishida and colleagues^29^ analyzed a cohort of 3,765 young adults, 83% of whom reported lifetime cannabis use. Kidney function was significantly lower in individuals that used cannabis for at least 5 years, but the authors did not observe a significant dose-response relationship. Nonetheless, this latter analysis was based on 5-year person-intervals, and only 3.4% (n=202 of 5,815) of the considered intervals corresponded to a consumption of >10 joints per year, thus suggesting a very low level of exposure throughout the cohort. Finally, Potukuchi et al^31^ did not observe increased risk of acute kidney injury and kidney disease progression in patients with advanced CKD who tested positive for cannabis in urine toxicology screening. Analysis of urine toxicology prevents report biases, but the test was only available in 2.2% of the investigated cohort (n=2,215 of 102,477 participants) and may not detect consumption after >1 week.

In this study, we found a significant association between daily and weekly cannabis use and kidney disease in a large and diverse cohort. Of note, we had quantitative data of cannabis consumption from >58,000 patients, which is more than the sum of all the patients analyzed in the abovementioned investigations. However, association studies cannot determine causation because they are limited by numerous biases; this is particularly true for studies investigating illegal substances. Among the most relevant biases, there is reverse causation (chronically ill people are more likely to use cannabis),^8^ the lack of distinction between medical versus recreational use, and selection/report bias. Our study highlights the latter issue: at first, we had to exclude almost 100,000 patients because of missing data about possible confounders. After exclusion, only 35.8% of the remaining participants (n=80,132 of 223,354) reported lifetime use of cannabis versus a national estimate of >50%.^1^ Finally, only 73.7% of this subgroup answered the quantitative questions. Additionally, it is likely that the available answers are further biased toward underreporting, thus increasing the risk of type II error. In this complex scenario, we suggest that the strongest available investigational tool is Mendelian randomization. Mendelian randomization assesses causality by comparing high- versus low-risk individuals and is independent of all the abovementioned biases.

Our Mendelian randomization analysis did not detect any increase in CKD risk in individuals at high risk of CUD. Thus, most likely there is no causal relationship between the 2 phenomena, and the association analysis is confounded. Our results were consistent across different Mendelian randomization analyses, including MR-PRESSO, which is designed to exclude horizontal pleiotropy thus limiting the possibility of a confounded association between the polymorphisms and the outcomes.

For BUN and cystatin-C eGFR, we found significant *P* values in a limited number of Mendelian randomization analyses but with a clinically irrelevant effect size (Table 2). These results do not contradict our conclusions, given the absence of effect. Nonetheless, if any effect should be postulated, the inconsistency among creatinine, BUN, and cystatin-C might be explained by the changes in diet and protein metabolism commonly observed in cannabis users (BUN),^32^ and possible effects of smoking as methodology of cannabis consumption (cystatin-C).^33^

Our Mendelian randomization analysis should be interpreted in the context of its limits. The genetic instruments were identified in a GWAS including primarily individuals of European ancestry; unfortunately, this bias is observed in the vast majority of available GWAS.^34^ Thus, the causal relationship assessed in this work may not be generalizable to other groups. Additionally, the cannabis GWAS used a dichotomous variable, and no quantitative parameters were reported (eg, dosage, frequency, length of exposure or medical vs recreational use). However, CUD identifies higher levels of consumption, thus strengthening our findings. Other authors have argued about possible limitations in using dichotomized exposure variables instead of continuous measures of risk in Mendelian randomization.^35^ Finally, our study did not distinguish among the different cannabinoids. Preclinical studies suggest that CB1 orchestrates tubular lipidic metabolism^6^^,^^36^ whereas CB2 may contribute to tubulo-interstitial fibrosis in CKD.^7^ To date, most of the available data on cannabis consumption refer to recreational use of nonregulated substances, which have a mix of different molecules. Due to its neurotropic proprieties, one could hypothesize that most recreational products are enriched in tetrahydrocannabinol, which acts mainly on CB1,^36^ but further mechanistic investigations are needed to decipher the specific effects of each receptor on kidneys.

In conclusion, this analysis provides both observational and Mendelian randomization analyses about cannabis and CKD. Although more frequent cannabis use was associated with CKD, Mendelian randomization analyses showed no relationship, indicating confounding in observational studies.

## Supplementary Material

Supplementary File (PDF) Fig S1, Item S1, Table S1-S2.
