# Supplementary material for: Cannabis Use and CKD: Epidemiological Associations and Mendelian Randomization
Source: Kidney Med. 2022 Dec 11;5(2):100582. doi: 10.1016/j.xkme.2022.100582 (PMC9879977; doi:10.1016/j.xkme.2022.100582)
Supplement: Supplementary File (PDF) — Fig S1, Item S1, Table S1-S2. [file mmc1.pdf]

Figure S1. Leave one out analysis for CKD.
As a sensitivity analysis, each SNP was sequentially excluded from the genetic instruments to identify outlier SNPs. Plots include odds ratios of each leave-one-out model for CAD using genetic instruments for cannabis use disorder (P <1x10^{-7}).

SNP **Odds Ratio (95% CI)**

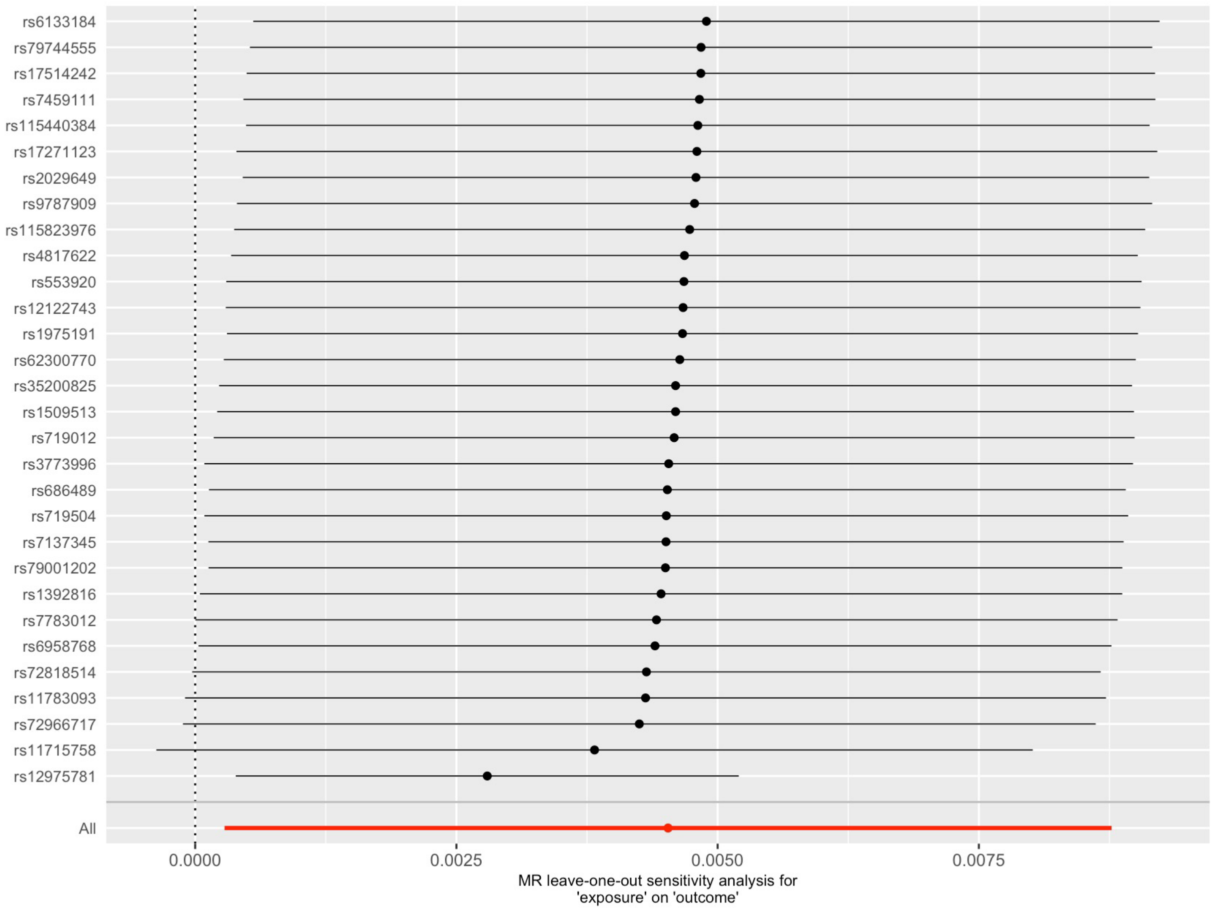

Item S1. Supplementary Methods

Questions in All of Us survey about cannabis consumption:

1. Have you smoked at least 100 cigarettes in your entire life? (There are 20 cigarettes in a pack.)?

   Answers: Yes, no
2. In your LIFETIME, which of the following substances have you ever used?

   Answers: Marijuana (cannabis, pot, grass, hash, weed, etc.). Other answers are included in the All of Us Lifestyle survey and omitted here for brevity.
3. In the PAST THREE MONTHS, how often have you used marijuana (cannabis, pot, grass, hash, etc.)?

   Answers: Never, once or twice, monthly, weekly, daily.
4. Are you covered by health insurance or some other kind of health care plan? Answers: Yes, no
5. What is the highest grade or year of school you completed?

   Answers: College graduate or advanced degree, 1-3 years of college, 12th or GED, Less than a high school degree

Supplementary Table 1. All of Us cohort summary (cannabis use frequency)**.** Demographics and clinical history summarized by 3-month cannabis use frequency in the All of Us cohort are provided. Only individuals who reported lifetime cannabis use and also reported cannabis use frequency in the three months prior to enrollment were included. Individuals who answered, "Prefer not to answer" or who skipped the cannabis use frequency question in the survey were excluded. Sex at birth, age, BMI, and cigarette use were ascertained using physical measurements and surveys administered at the time of enrollment in All of Us. Medical history was obtained from EHR data as well as self-reported survey data.

	**Three-month cannabis use frequency**							
**Variable**	**Never (N = 39,996)**	**Once or twice (N = 8,921)**	**Monthly (N = 2,133)**	**Weekly (N = 2,790)**	**Daily (N = 4,820)**	**Prefer not to answer (N = 865)**	**P Value**	
**Sex at birth, n (%)**								
**Male**	15,073 (38%)	3,688 (41%)	1,030 (48%)	1,380 (49%)	2,146 (45%)	397 (46%)	<0.001	
**Race, n (%)**								
**Asian**	1,137 (2.8%)	347 (3.9%)	55 (2.6%)	55 (2.0%)	38 (0.8%)	15 (1.7%)	<0.001	
**Black or African American**	7,402 (19%)	3,685 (41%)	1,185 (56%)	1,406 (50%)	2,772 (58%)	551 (64%)		
**More than one population**	892 (2.2%)	269 (3.0%)	54 (2.5%)	82 (2.9%)	150 (3.1%)	18 (2.1%)		
**Other**	618 (1.5%)	195 (2.2%)	42 (2.0%)	60 (2.2%)	118 (2.4%)	33 (3.8%)		
**White**	29,947 (75%)	4,425 (50%)	797 (37%)	1,187 (43%)	1,742 (36%)	248 (29%)		
**Age (years), Median (IQR)**	59 (41, 69)	44 (31, 59)	45 (32, 60)	45 (32, 60)	41 (32, 55)	47 (34, 59)	<0.001	
**BMI (kg/m2), Median (IQR)**	28 (24, 34)	27 (23, 33)	27 (23, 32)	27 (23, 32)	27 (23, 33)	27 (23, 32)	<0.001	
**Chronic kidney disease, n (%)**	1,458 (3.6%)	235 (2.6%)	58 (2.7%)	96 (3.4%)	161 (3.3%)	21 (2.4%)		
**Hypertension, n (%)**	10,818 (27%)	1,822 (20%)	437 (20%)	658 (24%)	1,116 (23%)	199 (23%)	<0.001	
**Type 2 diabetes, n (%)**	1,802 (4.5%)	321 (3.6%)	70 (3.3%)	95 (3.4%)	198 (4.1%)	30 (3.5%)	<0.001	
**Coronary artery disease, n (%)**	2,737 (6.8%)	372 (4.2%)	81 (3.8%)	128 (4.6%)	236 (4.9%)	37 (4.3%)	<0.001	
**Hyperlipidemia, n (%)**	9,359 (23%)	1,185 (13%)	243 (11%)	393 (14%)	638 (13%)	101 (12%)	<0.001	
**> 100 cigarettes smoked in lifetime, n (%)**								<0.001

**Yes**	16,932 (42%)	3,837 (43%)	1,068 (50%)	1,547 (55%)	3,044 (63%)	503 (58%)	290 (64%)	
**Has health insurance**								<0.001
**Yes**	38,531 (96%)	8,064 (90%)	1,865 (87%)	2,436 (87%)	4,178 (87%)	721 (83%)	399 (89%)	

Supplementary Table 2. Genetic instruments for cannabis use

Genome wide association study summary statistics for SNPs selected in cannabis use instruments. The main instruments were selected using a liberal instrument selection approach (P <5x10^{-6}) variants in a GWAS for CUD. The alternative instruments include independent SNPs meeting a genome wide significance threshold (P <5x10^{-8}) for CUD. SNPs were assigned to genes using dbSNP annotations.

Instrument	SNP	Gene name	Effect Allele	Non effect allele	Beta	SE	P value	F statistic
**Liberal P value threshold (5 x 10^{-6})**	rs115440384	None	T	C	0.274	0.0593	3.73E-06	21.38088
	rs115823976	CALCRL	A	G	-0.25	0.0518	2.21E-06	22.37034
	rs11715758	GNAI2	A	G	-0.09	0.0175	8.91E-08	28.54612
	rs11783093	EPHX2	T	C	-0.15	0.0244	2.68E-09	35.31477
	rs12122743	None	A	G	-0.08	0.0175	1.32E-06	23.3151
	rs12975781	IZUMO1	T	C	-0.08	0.0176	1.66E-06	22.83319
	rs1392816	PDE4B	T	C	-0.1	0.0184	6.14E-08	29.41883
	rs1509513	LOC107984390	A	G	0.086	0.0168	3.18E-07	26.02214
	rs17271123	None	T	G	0.128	0.0252	3.54E-07	25.96145
	rs17514242	TCF4	C	G	-0.09	0.0171	2.56E-07	26.48336
	rs1975191	None	T	G	-0.09	0.0192	3.20E-06	21.68066
	rs2029649	LOC105377408	T	C	0.079	0.0167	2.18E-06	22.54828
	rs35200825	None	T	C	-0.31	0.0662	2.31E-06	22.34062
	rs3773996	IL1RAP	A	G	-0.25	0.051	1.07E-06	23.81823
	rs4817622	None	A	G	0.078	0.0169	3.30E-06	21.52082
	rs553920	None	T	C	0.104	0.0198	1.60E-07	27.58902
	rs6133184	SLC23A2	T	C	0.126	0.0261	1.22E-06	23.4538
	rs62300770	None	T	C	0.09	0.019	2.18E-06	22.33806
	rs686489	None	A	G	0.084	0.0179	2.52E-06	22.23202
	rs6958768	MAGI2	A	C	0.11	0.0239	4.37E-06	21.02932

	rs7137345	PKP2	T	G	0.096	0.0199	1.49E-06	23.17527
	rs719012	SHTN1	T	C	0.096	0.0187	2.87E-07	26.40971
	rs719504	None	A	G	0.103	0.0192	9.00E-08	28.50003
	rs72818514	ATP10B	T	C	-0.18	0.0342	9.33E-08	28.56934
	rs72966717	None	T	G	-0.11	0.0226	1.07E-06	23.86279
	rs7459111	None	A	T	-0.09	0.0197	2.45E-06	22.23817
	rs7783012	FOXP2	A	G	0.101	0.0168	1.84E-09	36.143
	rs79001202	None	A	T	-0.09	0.0195	3.55E-06	21.49155
	rs79744555	None	T	C	0.141	0.03	2.63E-06	22.05868
	rs9787909	METTL15	A	C	0.114	0.0225	4.52E-07	25.53618
**Genome wide significant (5 x 10^{-8})**	rs4732724	EPHX2	C	G	-0.114	0.0196	6.5E-09	33.711
	rs7783012	FOXP2	A	G	0.101	0.0168	1.8E-09	36.143
